# Supplementary material for: Diverse Virulence Attributes of Pantoea alfalfae sp. nov. CQ10 Responsible for Bacterial Leaf Blight in Alfalfa Revealed by Genomic Analysis
Source: Int J Mol Sci. 2023 May 2;24(9):8138. doi: 10.3390/ijms24098138 (PMC10179099; doi:10.3390/ijms24098138)
Supplement: Supplementary file 1 [file ijms-24-08138-s001.zip › ijms-2330985-supplementary.pdf]

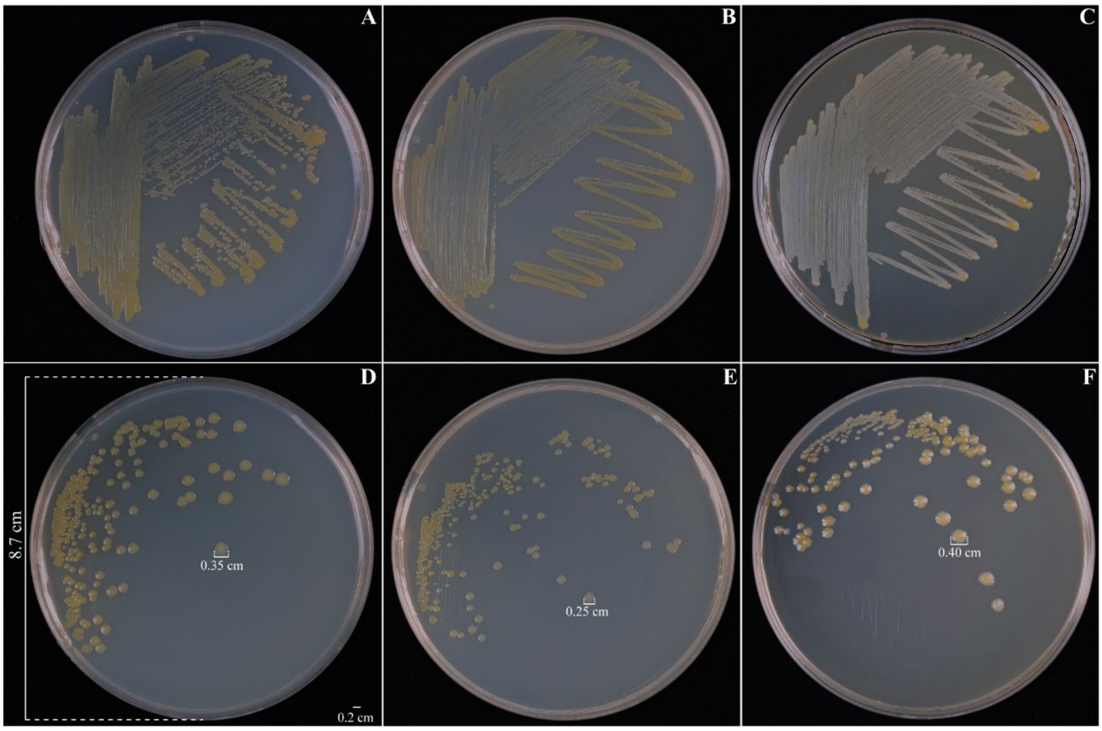

Figure S1 Photograph of colonial morphology of strain CQ10 grown on LB agar plates (A) NB agar plates (B) and TSB agar plates (C) for 48 h at 30°C. Single colony of strain CQ10 on (D) LB agar plates, (E) NB agar plates, and (F) TSB agar plates.

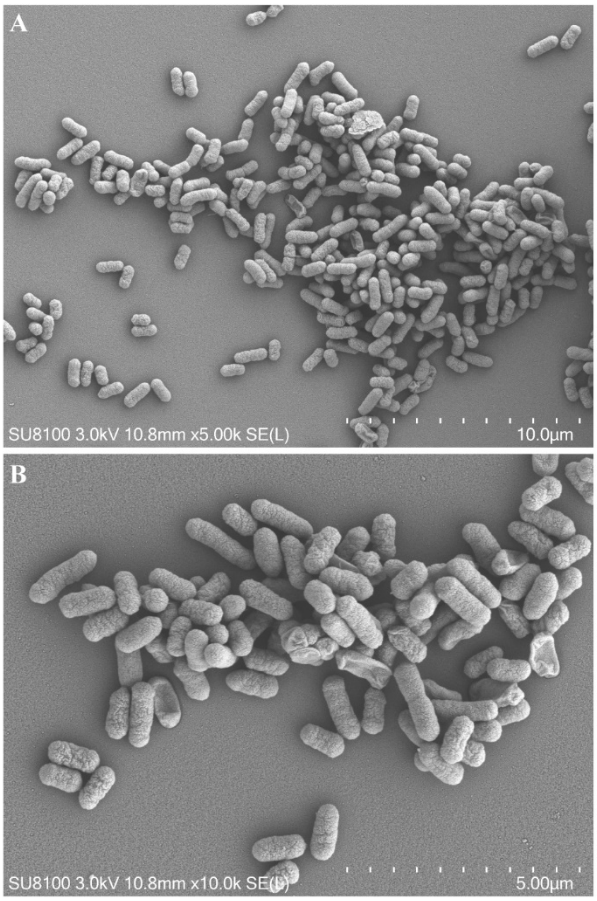

Figure S2 Scanning electron microscopy (SEM) imaging of strain CQ10. (A) Scale bar = 10.0 μM. (B) Scale bar = 5.0 μM.

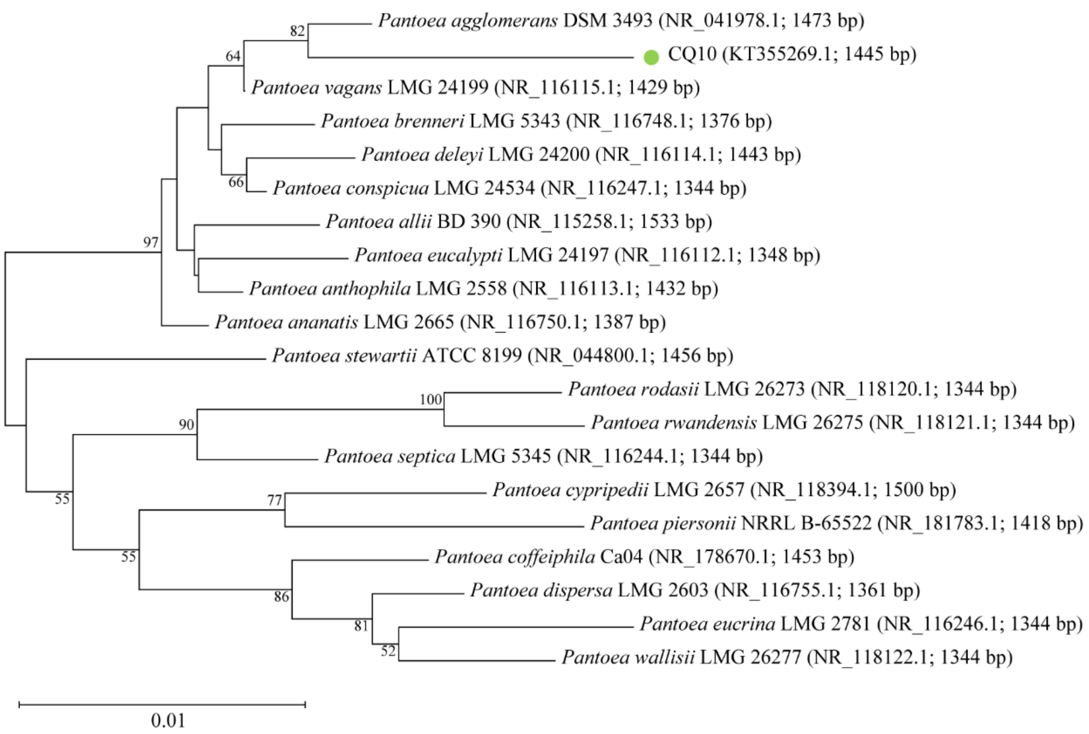

Figure S3 Neighbor-Joining phylogenetic tree based on 16S rRNA gene sequences showing the relationships between strain CQ10 and closely related 19 species (GenBank accession numbers and sequence lengths of the nucleotide sequences are given in parentheses). Numbers at branching points are bootstrap values > 50% (1000 replicates). Bar, 0.01 substitution per site. Green spot indicates strain CQ10. Evolutionary analyses were conducted in MEGA11.

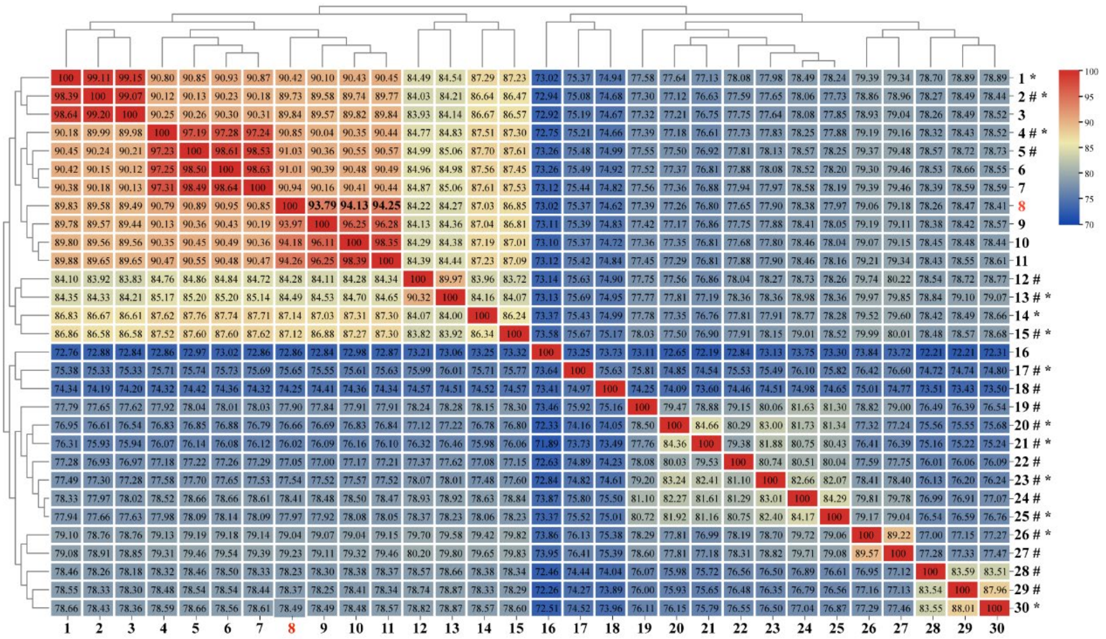

Figure S4 Heatmap showing ANI (average nucleotide identify) of representative *Pantoea* species. Numbers 1-30 represent *Pantoea* strains: [1]*P. hericii* JZB 2120024, [2]*P. eucalypti* LMG 24197, [3]*P. jilinensis* D25, [4]*P. pleuroti* JZB 2120015, [5]*P. agglomerans* FDAARGOS 1447, [6]*P. agglomerans* ASB05, [7]*P. agglomerans* DBM 3797, [8]CQ10, [9]*P. vagans* FDAARGOS_160, [10]*P. vagans* C9-1, [11]*P. vagans* PV989, [12]*P. brenneri* IIFCSG-B1, [13]*P. conspicua* LMG 24534, [14]*P. anthophila* LMG 2558, [15]*P. deleyi* LMG 24200, [16]*P. sesami* Si-M154, [17]*P. alhagi* LTYR-11Z, [18]*P. coffeiphila* 1480, [19]*P. eucrina* XL123, [20]*P. rodasii* DSM 26611, [21]*P. rwandensis* LMG 26275, [22]*P. cypripedii* WS4375, [23]*P. endophytica* 596, [24]*P. dispersa* Lsch, [25]*P. wallisii* LMG 26277, [26]*P. latae* AS1, [27]*P. septica* MGYG-HGUT-02423, [28]*P. stewartii* ZJ-FGZX1, [29]*P. allii* PNA 200-10, [30]*P. ananatis* LMG 2665. # Represents reference genome in the NCBI genome database, * represents type strain.

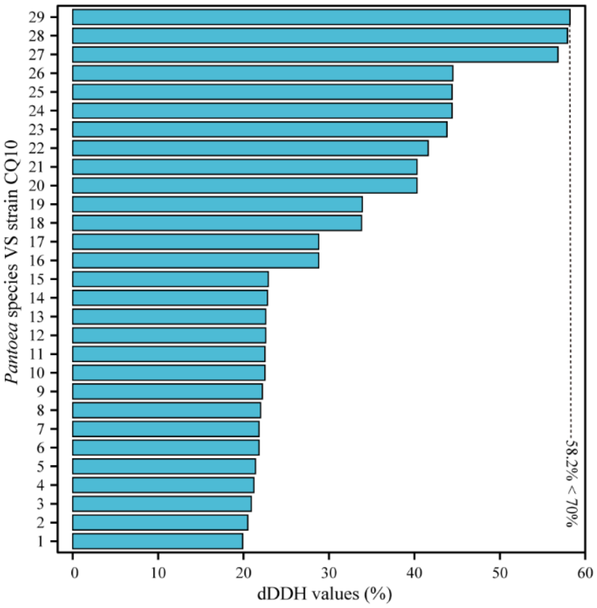

Figure S5 Analysis of the digital DNA-DNA hybridisation (dDDH, species cut-off ≥ 70%) values of strain CQ10 versus 29 *Pantoea* species. Numbers 1-29 indicates: [1]*P. sesami* Si-M154, [2]*P. coffeiphila* 1480, [3]*P. alhagi* LTYR-11Z, [4]*P. rwandensis* LMG 26275, [5]*P. rodasii* DSM 26611, [6]*P. endophytica* 596, [7]*P. eucrina* XL123, [8]*P. cypripedii* WS4375, [9]*P. wallisii* LMG 26277, [10]*P. allii* PNA 200-10, [11]*P. ananatis* LMG 2665, [12]*P. dispersa* Lsch, [13]*P. stewartii* ZJ-FGZX1, [14]*P. latae* AS1, [15]*P. septica* MGYG-HGUT-02423, [16]*P. brenneri* IIFCSG-B1, [17]*P. conspicua* LMG 24534, [18]*P. deleyi* LMG 24200, [19]*P. anthophila* LMG 2558, [20]*P. eucalypti* LMG 24197, [21]*P. jilinensis* D25, [22]*P. hericii* JZB 2120024, [23]*P. pleuroti* JZB 2120015, [24]*P. agglomerans* ASB05, [25]*P. agglomerans* DBM 3797, [26]*P. agglomerans* FDAARGOS 1447, [27]*P. vagans* FDAARGOS_160, [28]*P. vagans* C9-1, [29]*P. vagans* PV989.

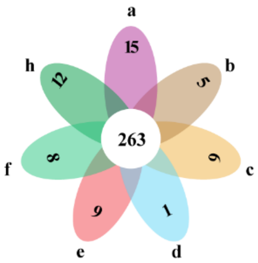

Top five virulence factors shared by strain CQ10 and six closely related species

Virulence factors	Function	Number of matched genes in *Pantoea* strains						
		a	b	c	d	e	f	g
peritrichous flagella	motility	28	29	32	30	29	26	28
pyoverdine	iron acquisition	26	26	38	33	35	30	24
HitABC	iron acquisition	24	22	20	18	20	23	22
FbpABC	iron acquisition	23	28	29	28	26	28	28
CdpA	regulation	17	19	21	18	18	17	16

a CQ10 (GCF_019880205.1)
b *P. vagans* C9-1 (GCF_000148935.1)
c *P. agglomerans* ASB05 (GCF_009762725.1)
d *P. agglomerans* FDAARGOS 1447 (GCF_019048385.1)
e *P. agglomerans* DBM 3797 (GCF_020783495.1)
f *P. vagans* PV989 (GCF_003032455.1)
g *P. vagans* FDAARGOS_160 (GCF_001558735.2)

Figure S6 Petal diagram of virulence factors of *Pantoea* strains. The numbers of common virulence factors in seven *Pantoea* strains was represented in the center and unique virulence factors in the petals. Latest Refseq accession numbers for strains are indicated in the legend in parentheses.

Table S1 General genome features of strain CQ10

Feature	Size (bp)	GC content (%)	CDS	tRNAs	rRNAs	misc_RNAs	tmRNAs
Chromosome	3,973,606	55.45	3591	78	22	82	1
Plasmid 1	235,469	51.92	234	0	0	1	0
Plasmid 2	160,986	52.33	136	0	0	0	0
Plasmid 3	143,561	52.57	140	0	0	1	0
Plasmid 4	137,838	55.59	120	0	0	2	0
Plasmid 5	89,114	50.72	99	3	0	2	0
Plasmid 6	4,424	40.71	5	0	0	0	0
Plasmid 7	2,709	62.24	4	0	0	0	0
Plasmid 8	2,495	47.82	1	0	0	0	0

Table S2 COG classification of CQ10 genes

Category code	Category name	CQ10 gene number	Percentage (%)
E	Amino acid transport and metabolism	295	11.29%
G	Carbohydrate transport and metabolism	234	8.96%
J	Translation, ribosomal structure and biogenesis	210	8.04%
M	Cell wall/membrane/envelope biogenesis	190	7.27%
C	Energy production and conversion	181	6.93%
P	Inorganic ion transport and metabolism	173	6.62%
K	Transcription	151	5.78%
R	General function prediction only	150	5.74%
H	Coenzyme transport and metabolism	143	5.47%
S	Function unknown	134	5.13%
T	Signal transduction mechanisms	116	4.44%
L	Replication, recombination and repair	113	4.33%
O	Posttranslational modification, protein turnover, chaperones	106	4.06%
F	Nucleotide transport and metabolism	89	3.41%
I	Lipid transport and metabolism	80	3.06%
N	Cell motility	78	2.99%
V	Defense mechanisms	56	2.14%
D	Cell cycle control, cell division, chromosome partitioning	43	1.65%
Q	Secondary metabolites biosynthesis, transport and catabolism	38	1.45%
U	Intracellular trafficking, secretion, and vesicular transport	27	1.03%
W	Extracellular structures	2	0.08%
X	Mobilome: prophages, transposons	2	0.08%
A	RNA processing and modification	1	0.04%

Table S3 The ANI and dDDH values between strain CQ10 and other *Pantoea* species

Species	ANI (%)	dDDH (%)
Pantoea vagans PV989	94.25	58.2
Pantoea vagans C9-1	94.13	57.9
Pantoea vagans FDAARGOS_160	93.79	56.8
Pantoea agglomerans ASB05	90.95	44.4
Pantoea agglomerans FDAARGOS 1447	90.89	44.5
Pantoea agglomerans DBM 3797	90.85	44.4
Pantoea pleuroti JZB 2120015	90.79	43.8
Pantoea hericii JZB2120024	89.83	41.6
Pantoea eucalypti LMG 24197	89.58	40.3
Pantoea jilinensis D25	89.49	40.3
Pantoea anthophila LMG 2558	87.03	33.9
Pantoea deleyi LMG 24200	86.85	33.8
Pantoea conspicua LMG 24534	84.27	28.8
Pantoea brenneri IIFCSG-B1	84.22	28.8
Pantoea septica MGYG-HGUT-02423	79.18	22.9
Pantoea latae AS1	79.06	22.8
Pantoea allii PNA 200-10	78.47	22.5
Pantoea ananatis LMG 2665	78.41	22.5
Pantoea dispersa Lsch	78.38	22.6
Pantoea stewartii ZJ-FGZX1	78.26	22.6
Pantoea wallisii LMG 26277	77.97	22.2
Pantoea endophytica 596	77.9	21.8
Pantoea cypripedii WS4375	77.65	22
Pantoea eucrina XL123	77.39	21.8
Pantoea rodasii DSM 26611	77.26	21.4
Pantoea rwandensis LMG 26275	76.8	21.2
Pantoea alhagi LTYR-11Z	75.37	20.9
Pantoea coffeiphila 1480	74.62	20.5
Pantoea sesami Si-M154	73.02	19.9

Table S4 Correlation analysis of physiological indices in roots, stems, and leaves of alfalfa

PI	RPI	Correlation	Absolute value of r	Significance	SPI	Correlation	Absolute value of r	Significance	LPI	Correlation	Absolute value of r	Significance
SOD	**PAL**	**-**	**0.83**	*	**PAL**	**-**	**0.94**	**	**PAL**	+	**0.94**	**
	MDA	-	0.89	*	MDA	-	0.94	**	CAT	-	0.89	*
	**APX**	+	**0.94**	**	**POD**	+	**0.83**	*	**POD**	-	**0.83**	*
	SP	+	0.83	*	PPO	-	1.00	***	SS	+	1.00	***
CAT	PPO	+	0.83	*	PPO	-	0.94	**	SOD	-	0.89	*
	**SS**	+	**0.94**	**	**SP**	+	**0.83**	*	**SS**	-	**0.89**	*
	/	/	/	/	PAL	-	0.83	*	PAL	-	0.94	**
	/	/	/	/	MDA	-	0.89	*	POD	+	0.89	*
APX	**POD**	+	**0.89**	*	POD	+	0.89	*	MDA	+	0.94	**
	SOD	+	0.94	**	SP	+	0.94	**	SP	-	0.94	**
	/	/	/	/	/	/	/	/	PPO	-	0.83	*
POD	APX	+	0.89	*	APX	+	0.89	*	CAT	+	0.89	*
	/	/	/	/	SOD	+	0.83	*	SOD	-	0.83	*
	/	/	/	/	SP	+	0.94	**	SS	-	0.83	*
	/	/	/	/	PPO	-	0.83	*	PAL	-	0.94	**
MDA	SOD	-	0.89	*	SOD	-	0.94	**	APX	+	0.94	**
	**SP**	**-**	**0.89**	*	**CAT**	**-**	**0.89**	*	**SP**	**-**	**1.00**	***
	PAL	+	0.83	*	/	/	/	/	/	/	/	/
PAL	SOD	-	0.83	*	SOD	-	0.94	**	SOD	+	0.94	**
	MDA	+	0.83	*	CAT	-	0.83	*	CAT	-	0.94	**
	SP	-	0.89	*	/	/	/	/	POD	-	0.94	**
	/	/	/	/	/	/	/	/	SS	+	0.94	**

Table S4 Correlation analysis of physiological indices in roots, stems, and leaves of alfalfa (continued)

PI	RPI	Correlation	Absolute value of r	Significance	SPI	Correlation	Absolute value of r	Significance	LPI	Correlation	Absolute value of r	Significance
PPO	CAT	+	0.83	*	CAT	-	0.94	**	APX	-	0.83	*
	/	/	/	/	SOD	-	1.00	***	/	/	/	/
	/	/	/	/	POD	-	0.83	*	/	/	/	/
SS	CAT	+	0.94	**	SP	-	0.83	*	CAT	-	0.89	*
	/	/	/	/	/	/	/	/	SOD	+	1.00	***
	/	/	/	/	/	/	/	/	PAL	+	0.94	**
	/	/	/	/	/	/	/	/	POD	-	0.83	*
SP	SOD	+	0.83	*	APX	+	0.94	**	APX	-	0.94	**
	MDA	-	0.89	*	POD	+	0.94	**	MDA	-	1.00	***
	PAL	-	0.89	*	**CAT**	+	**0.83**	*	/	/	/	/
	/	/	/	/	**SS**	-	**0.83**	*	/	/	/	/

*PI, physiological indices; RPI, root physiological indices; SPI, stem physiological indices; LPI, leaf physiological indices. + represent positive correlation, - represent negative correlation, r represent correlation coefficient. *, P < 0.05; **, P < 0.01; ***, P < 0.001.*

Table S5 COG classification of core genes in each orthologous group

COG Categories	COG Subcategories	Category code	Number of Matched Genes	Percentage (%)
Poorly characterized	Function unknown	S	703	19.41
Metabolism	Amino acid transport and metabolism	E	325	8.98
	Inorganic ion transport and metabolism	P	316	8.73
	Carbohydrate transport and metabolism	G	278	7.68
	Energy production and conversion	C	214	5.91
	Coenzyme transport and metabolism	H	193	5.33
	Nucleotide transport and metabolism	F	98	2.71
	Lipid transport and metabolism	I	96	2.65
	Secondary metabolites biosynthesis, transport and catabolism	Q	56	1.55
Cellular processes and signaling	Cell wall/membrane/envelope biogenesis	M	223	6.16
	Signal transduction mechanisms	T	140	3.87
	Posttranslational modification, protein turnover, chaperones	O	98	2.71
	Cell motility	N	91	2.51
	Intracellular trafficking, secretion, and vesicular transport	U	86	2.38
	Cell cycle control, cell division, chromosome partitioning	D	50	1.38
	Defense mechanisms	V	38	1.05
Information storage and processing	Transcription	K	303	8.37
	Translation, ribosomal structure and biogenesis	J	183	5.05
	Replication, recombination and repair	L	130	3.59

Table S6 COG classification of strain unique genes in each orthologous group

COG Categories	COG Subcategories	Category code	a		b		c		d	
			Num.	Pct.	Num.	Pct.	Num.	Pct.	Num.	Pct.
Poorly characterized	Function unknown	S	1	3.8	7	19.4	9	18.4	18	27.3
Metabolism	Amino acid transport and metabolism	E	1	3.8	1	2.8	1	2.0	3	4.5
	Inorganic ion transport and metabolism	P	3	11.5	0	0.0	0	0.0	2	3.0
	Carbohydrate transport and metabolism	G	2	7.7	3	8.3	1	2.0	3	4.5
	Energy production and conversion	C	1	3.8	0	0.0	2	4.1	2	3.0
	Coenzyme transport and metabolism	H	2	7.7	0	0.0	0	0.0	3	4.5
	Nucleotide transport and metabolism	F	1	3.8	3	8.3	1	2.0	1	1.5
	Lipid transport and metabolism	I	1	3.8	1	2.8	1	2.0	6	9.1
	Secondary metabolites biosynthesis, transport and catabolism	Q	2	7.7	1	2.8	1	2.0	5	7.6
Cellular processes and signaling	Cell wall/membrane/envelope biogenesis	M	2	7.7	2	5.6	3	6.1	4	6.1
	Signal transduction mechanisms	T	1	3.8	1	2.8	1	2.0	2	3.0
	Posttranslational modification, protein turnover, chaperones	O	2	7.7	0	0.0	0	0.0	2	3.0
	Cell motility	N	1	3.8	1	2.8	1	2.0	0	0.0
	Intracellular trafficking, secretion, and vesicular transport	U	2	7.7	0	0.0	2	4.1	0	0.0
	Cell cycle control, cell division, chromosome partitioning	D	1	3.8	1	2.8	1	2.0	2	3.0
	Defense mechanisms	V	0	0.0	2	5.6	7	14.3	2	3.0
Information storage and processing	Transcription	K	2	7.7	7	19.4	9	18.4	6	9.1
	Translation, ribosomal structure and biogenesis	J	0	0.0	0	0.0	1	2.0	0	0.0
	Replication, recombination and repair	L	1	3.8	6	16.7	8	16.3	5	7.6

Table S6 COG classification of strain unique genes in each orthologous group (continued)

COG Categories	COG Subcategories	Category code	e		f		g		h	
			**Num.**	**Pct.**	**Num.**	**Pct.**	**Num.**	**Pct.**	**Num.**	**Pct.**
Poorly characterized	Function unknown	S	26	22.6	55	41.0	51	35.4	69	36.7
Metabolism	Amino acid transport and metabolism	E	11	9.6	8	6.0	2	1.4	16	8.5
	Inorganic ion transport and metabolism	P	8	7.0	8	6.0	0	0.0	13	6.9
	Carbohydrate transport and metabolism	G	10	8.7	12	9.0	5	3.5	8	4.3
	Energy production and conversion	C	2	1.7	3	2.2	1	0.7	6	3.2
	Coenzyme transport and metabolism	H	3	2.6	3	2.2	0	0.0	4	2.1
	Nucleotide transport and metabolism	F	4	3.5	1	0.7	2	1.4	2	1.1
	Lipid transport and metabolism	I	2	1.7	3	2.2	8	5.6	3	1.6
	Secondary metabolites biosynthesis, transport and catabolism	Q	4	3.5	3	2.2	2	1.4	1	0.5
Cellular processes and signaling	Cell wall/membrane/envelope biogenesis	M	2	1.7	2	1.5	15	10.4	8	4.3
	Signal transduction mechanisms	T	6	5.2	7	5.2	0	0.0	10	5.3
	Posttranslational modification, protein turnover, chaperones	O	2	1.7	2	1.5	6	4.2	4	2.1
	Cell motility	N	4	3.5	2	1.5	5	3.5	5	2.7
	Intracellular trafficking, secretion, and vesicular transport	U	4	3.5	3	2.2	13	9.0	8	4.3
	Cell cycle control, cell division, chromosome partitioning	D	1	0.9	1	0.7	2	1.4	2	1.1
	Defense mechanisms	V	2	1.7	3	2.2	9	6.3	2	1.1
Information storage and processing	Transcription	K	9	7.8	14	10.4	12	8.3	20	10.6
	Translation, ribosomal structure and biogenesis	J	4	3.5	0	0.0	1	0.7	0	0.0
	Replication, recombination and repair	L	11	9.6	4	3.0	10	6.9	7	3.7

Num. represent number fo matched core genes. Pct. represent percentage (%). a, Pantoea alfalfae CQ10. b, Pantoea agglomerans FDAARGOS 1447. c, Pantoea agglomerans DBM 3797. d, Pantoea agglomerans ASB05. e, Pantoea vagans C9-1. f, Pantoea pleuroti JZB 2120015. g, Pantoea vagans PV989. h, Pantoea vagans FDAARGOS_160.

Table S7 The collinearity analysis of the strain CQ10 genome and the closest *Pantoea* genomes

Query Species	Query sequence location	Subject sequence location	Number of collinearity region	Percentage of total collinearity region (%)
Pantoea agglomerans DBM 3797	Chromosome	Chromosome_CQ10	2199	93.66
	Plasmid pPA_DBM3797_1		60	
	Plasmid pPA_DBM3797_2		1	
	Chromosome	Plasmid p1_CQ10	3	2.20
	Plasmid pPA_DBM3797_1		49	
	Plasmid pPA_DBM3797_2		1	
	Chromosome	Plasmid p2_CQ10	2	1.28
	Plasmid pPA_DBM3797_2		29	
	Chromosome	Plasmid p3_CQ10	8	1.28
	Plasmid pPA_DBM3797_1		22	
	Plasmid pPA_DBM3797_2		1	
	Chromosome	Plasmid p4_CQ10	21	1.37
	Plasmid pPA_DBM3797_1		12	
	Chromosome	Plasmid p5_CQ10	1	0.08
	Plasmid pPA_DBM3797_2		1	
	Chromosome	Plasmid p6_CQ10	1	0.04
	Plasmid pPA_DBM3797_1	Plasmid p8_CQ10	2	0.08
Pantoea agglomerans FDAARGOS 1447	Chromosome	Chromosome_CQ10	2190	93.89
	Plasmid unnamed1		2	
	Plasmid unnamed2		53	
	Chromosome	Plasmid p1_CQ10	5	2.17
	Plasmid unnamed1		1	
	Plasmid unnamed2		46	

Table S7 The collinearity analysis of the strain CQ10 genome and the closest *Pantoea* genomes (continued)

Query Species	Query sequence location	Subject sequence location	Number of collinearity region	Percentage of total collinearity region
Pantoea agglomerans FDAARGOS 1447	Chromosome	Plasmid p2_CQ10	3	1.30
	Plasmid unnamed1		28	
	Chromosome	Plasmid p3_CQ10	7	1.30
	Plasmid unnamed1		2	
	Plasmid unnamed2		22	
	Chromosome	Plasmid p4_CQ10	17	1.17
	Plasmid unnamed2		11	
	Plasmid unnamed1	Plasmid p5_CQ10	2	0.13
	Plasmid unnamed2		1	
	Plasmid unnamed2	Plasmid p8_CQ10	1	0.04
Pantoea agglomerans ASB05	Chromosome	Chromosome_CQ10	2162	93.43
	Plasmid pASB05p1		9	
	Plasmid pASB05p2		2	
	Plasmid pASB05p3		2	
	Chromosome	Plasmid p1_CQ10	3	2.19
	Plasmid pASB05p1		47	
	Plasmid pASB05p2		1	
	Chromosome	Plasmid p2_CQ10	1	1.29
	Plasmid pASB05p2		29	
	Chromosome	Plasmid p3_CQ10	8	1.25
	Plasmid pASB05p1		19	
	Plasmid pASB05p2		1	
	Plasmid pASB05p3		1	

Table S7 The collinearity analysis of the strain CQ10 genome and the closest *Pantoea* genomes (continued)

Query Species	Query sequence location	Subject sequence location	Number of collinearity region	Percentage of total collinearity region
Pantoea agglomerans ASB05	Chromosome	Plasmid p4_CQ10	21	1.33
	Plasmid pASB05p1		10	
	Plasmid pASB05p2	Plasmid p5_CQ10	3	0.47
	Plasmid pASB05p3		8	
	Plasmid pASB05p1	Plasmid p8_CQ10	1	0.04
Pantoea vagans PV989	Chromosome	Chromosome_CQ10	2108	94.75
	Plasmid pPV989-167		3	
	Plasmid pPV989-508		70	
	Plasmid pPV989-94		1	
	Chromosome	Plasmid p1_CQ10	2	1.13
	Plasmid pPV989-508		24	
	Chromosome	Plasmid p2_CQ10	1	1.17
	Plasmid pPV989-167		25	
	Plasmid pPV989-508		1	
	Chromosome	Plasmid p3_CQ10	3	1.22
	Plasmid pPV989-167		1	
	Plasmid pPV989-508		22	
	Plasmid pPV989-94		2	
	Chromosome	Plasmid p4_CQ10	22	1.26
	Plasmid pPV989-508		7	
	Plasmid pPV989-94	Plasmid p5_CQ10	9	0.39
	Plasmid pPV989-508	Plasmid p8_CQ10	2	0.09

Table S7 The collinearity analysis of the strain CQ10 genome and the closest *Pantoea* genomes (continued)

Query Species	Query sequence location	Subject sequence location	Number of collinearity region	Percentage of total collinearity region
Pantoea vagans FDAARGOS_160	Chromosome	Chromosome_CQ10	2112	94.10
	Plasmid unnamed2		9	
	Chromosome	Plasmid p1_CQ10	1	1.29
	Plasmid unnamed1		1	
	Plasmid unnamed2		27	
	Chromosome	Plasmid p2_CQ10	3	1.46
	Plasmid unnamed2		1	
	Plasmid unnamed3		29	
	Chromosome	Plasmid p3_CQ10	4	1.29
	Plasmid unnamed1		1	
	Plasmid unnamed2		24	
	Chromosome	Plasmid p4_CQ10	12	0.98
	Plasmid unnamed2		10	
	Plasmid unnamed1	Plasmid p5_CQ10	17	0.80
	Plasmid unnamed3		1	
	Plasmid unnamed2	Plasmid p8_CQ10	2	0.09
Pantoea vagans C9-1	Chromosome	Chromosome_CQ10	1927	94.32
	Plasmid pPag2		3	
	Plasmid pPag3		64	
	Chromosome	Plasmid p1_CQ10	1	1.32
	Plasmid pPag2		2	
	Plasmid pPag3		25	

Table S7 The collinearity analysis of the strain CQ10 genome and the closest *Pantoea* genomes (continued)

Query Species	Query sequence location	Subject sequence location	Number of collinearity region	Percentage of total collinearity region
Pantoea vagans C9-1	Chromosome	Plasmid p2_CQ10	1	1.23
	Plasmid pPag1		24	
	Plasmid pPag3		1	
	Chromosome	Plasmid p3_CQ10	3	1.14
	Plasmid pPag2		1	
	Plasmid pPag3		20	
	Chromosome	Plasmid p4_CQ10	20	1.28
	Plasmid pPag3		7	
	Plasmid pPag2	Plasmid p5_CQ10	12	0.57
	Chromosome	Plasmid p6_CQ10	1	0.05
	Plasmid pPag3	Plasmid p8_CQ10	2	0.09

Table S8 Analysis of unique virulence factors for strain CQ10 and other species of genus *Pantoea*

Species	Location of the query sequence	Start	End	VFs	Function
CQ10	chromosome	802581	803135	F1C fimbriae	Biofilm
	chromosome	804018	806624	Type 1 fimbriae	Biofilm
	chromosome	806664	807623	Type 3 fimbriae	Biofilm
	chromosome	1197506	1199890	F1 antigen	Immune modulation
	chromosome	1199944	1200651	Haemagglutinating pili	Adherence
	chromosome	1670197	1671402	Flagella	Motility
	chromosome	2065798	2066361	Type 1 fimbriae	Biofilm
	chromosome	2066374	2067438	Type 1 fimbriae	Biofilm
	chromosome	2071495	2072052	Type 1 fimbriae	Biofilm
	plasmid_1	84815	86050	CAI-1	Biofilm
	plasmid_1	186711	188699	Flagella	Motility
	plasmid_3	74875	76035	MtrCDE	Antimicrobial activity/Competitive advantage
	plasmid_4	76819	77292	Polar flagella	Motility
	plasmid_5	42803	44905	Polar flagella	Motility
	plasmid_6	1864	2793	Capsule	Immune modulation
Pantoea agglomerans ASB05	chromosome	443659	444432	Exe T2SS	Effector delivery system
	chromosome	619149	627734	CβG	Immune modulation
	plasmid_2	160988	162469	Pyoverdine	Nutritional/Metabolic factor
	plasmid_2	169832	170695	Phenazines biosynthesis	-
	plasmid_2	170695	172524	Phenazines biosynthesis	-
	plasmid_2	172592	173221	Phenazines biosynthesis	-
	plasmid_2	173355	173831	Phenazines biosynthesis	-
	plasmid_3	39266	39889	Polar flagella	Motility
	plasmid_3	55328	56044	TcpC	Immune modulation

Table S8 Analysis of unique virulence factors for strain CQ10 and other species of genus *Pantoea* (continued)

Species	Location of the query sequence	Start	End	VFs	Function
Pantoea agglomerans DBM 3797	chromosome	2440903	2443026	TTSS	Effector delivery system
	chromosome	2447277	2447930	TTSS	Effector delivery system
	chromosome	2447938	2448198	TTSS	Effector delivery system
	chromosome	2448198	2449013	TTSS	Effector delivery system
	chromosome	2449010	2450095	TTSS	Effector delivery system
	chromosome	3059010	3059513	Afimbrial adhesin, AfaE-VIII	-
	chromosome	3059529	3062093	Afimbrial adhesin, AFA-I, mannose-resistant adhesin	-
	chromosome	3062090	3062797	Afimbrial adhesin, AfaE-VIII	-
	plasmid_1	147372	148892	Lateral flagella	Motility
Pantoea agglomerans FDAARGOS 1447	chromosome	3380417	3381889	Capsule	Immune modulation
Pantoea vagans C9-1	chromosome	2057889	2058782	Rhamnolipid biosynthesis	-
	plasmid_1	81144	82571	Sat	Effector delivery system
	plasmid_2	33286	35376	Aerobactin	Nutritional/Metabolic factor
	plasmid_2	104268	106007	pyoverdine	Nutritional/Metabolic factor
	plasmid_2	109232	111055	LPS	Immune modulation
Pantoea vagans FDAARGOS_160	chromosome	581583	584105	F1C fimbriae	Biofilm
	chromosome	3089419	3091341	SpA	Exotoxin
	chromosome	3555820	3556851	Shu	Nutritional/Metabolic factor
	chromosome	3539544	3540539	Tap type IV pili	Adherence
	plasmid_1	37370	37903	LEE encoded T3SS	-
	plasmid_3	32619	33635	Yersiniabactin	Nutritional/Metabolic factor
	plasmid_3	70496	72649	TTSS	Effector delivery system
	plasmid_3	76831	77484	TTSS	Effector delivery system

Table S8 Analysis of unique virulence factors for strain CQ10 and other species of genus *Pantoea* (continued)

Species	Location of the query sequence	Start	End	VFs	Function
Pantoea vagans FDAARGOS_160	plasmid_3	77492	77752	T3SS1	Effector delivery system
	plasmid_3	77755	78552	TTSS	Effector delivery system
	plasmid_3	78554	79636	T3SS	Effector delivery system
	plasmid_3	96830	100786	Pic	Effector delivery system
Pantoea vagans PV989	chromosome	3430499	3432181	Flagella	Motility
	plasmid_167	69950	73909	Tsh	Effector delivery system
	plasmid_94	39010	41196	Dot/Icm T4SS	Effector delivery system
	plasmid_94	64994	66160	Dot/Icm T4SS	Effector delivery system
	plasmid_94	67592	68986	Type IV pili	Adherence
	plasmid_94	70805	71920	Type IV pili	Adherence
	plasmid_94	71913	73436	Type IV pili	Adherence
	plasmid_94	75320	76951	Type IV pili	Adherence

Table S9 Pathogen-host interaction (PHI) analysis of strain CQ10

PHI categories	Genes	Numbers of matched genes in strain CQ10
increased virulence (hypervirulence)	raxB	97
	kdpE	56
	ShvR (BCAS0225)	37
	rcsC11	36
	bcam1160	34
	bcam1161	34
	MorA	34
	rpfR (bcam0580)	33
	cdpA (bcal1069)	30
	tcrX	29
	luxO	28
	gigX4	26
	tlp2 (Cjj81176_0180)	26
	gigX5	24
	THR	24
	AlHK1	23
	bcal1635	23
	CovR	23
	PmrA	23
	QseB	23
	atsR	22
	ECA2008	22
	gcpA (Dda3937_03858)	22
	tcrY	22

Table S9 Pathogen-host interaction (PHI) analysis of strain CQ10 (continued)

PHI categories	Genes	Numbers of matched genes in strain CQ10
increased virulence (hypervirulence)	bcal0430	21
	bcam1670	21
	bcam2256	21
	bcam2836	21
	ccpE	21
	kdpD	21
	raxR	21
	bcal0621	20
	bcal1975	20
	bcal2852	20
	bcam0748	20
	bcam1554	20
	bcam2822	20
	bcas0398	20
	BphP1	20
	cgsB	20
	dgcA	20
	GdpX1	20
	bcal0652	19
	bcam0158	19
	bcam2426	19
	ssrB	19
	bcal1100	18
	CovRS	18

Table S9 Pathogen-host interaction (PHI) analysis of strain CQ10 (continued)

PHI categories	Genes	Numbers of matched genes in strain CQ10
increased virulence (hypervirulence)	CovS	18
	vraR	16
	trcS	15
	BfiS	13
	BfvR (YPO1737)	13
	glbC	13
	PhoQ	13
	raxH	13
	App	11
	dosR (devR)	11
	Opp	11
	RpiRc	11
	bcas0263	10
	LacR	10
	GntR	8
	hns	8
	HxfA (PD_2118) (pspA)	8
	VEDA_05196	8
	Lmo0866	7
	Lmo1246	6
	Lmo1450	6
	Lmo1722	6
	lon	6
	lrp	6

Table S9 Pathogen-host interaction (PHI) analysis of strain CQ10 (continued)

PHI categories	Genes	Numbers of matched genes in strain CQ10
increased virulence (hypervirulence)	PKS	6
	raxQ	6
	rpfF	6
	spxB	6
	AOL_s00215g283	5
	EAMY_3025	5
	Ohmm	5
	pchD	5
	phuR	5
	VEDA_05198	5
	bcal2749	4
	EAMY_3224	4
	esaN	4
	Rv0462 (lpdC)	4
	Rv1773c	4
	Rv3213c	4
	sidN	4
	ymoA	4
	AS87_RS09170 (bioF)	3
	bcas0378	3
	BfeA (PD_1198)	3
	Can2	3
	CD3284	3
	EAMY_3005	3

Table S9 Pathogen-host interaction (PHI) analysis of strain CQ10 (continued)

PHI categories	Genes	Numbers of matched genes in strain CQ10
increased virulence (hypervirulence)	EAMY_3222	3
	gigX10	3
	htrA	3
	LysX	3
	mgtC	3
	PA3242	3
	pecS	3
	pknH	3
	ptsI	3
	raxC	3
	Rho	3
	UgeB	3
	adeN	2
	BcFKBP12	2
	bcsA	2
	CdpR	2
	crc1	2
	EAMY_3004	2
	EAMY_3010	2
	EAMY_3011	2
	EAMY_3012	2
	EAMY_3015	2
	EAMY_3020	2
	EAMY_3023	2

Table S9 Pathogen-host interaction (PHI) analysis of strain CQ10 (continued)

**PHI categories**	**Genes**	**Numbers of matched genes in strain CQ10**
increased virulence (hypervirulence)	EAMY_3028	2
	EAMY_3227	2
	EF-P	2
	feoB	2
	fliC	2
	fljB	2
	gigX1	2
	katE	2
	NTH1	2
	NTH2	2
	Pat	2
	picC	2
	PrsA	2
	RsmA	2
	Rv2220 (glnA1)	2
	S5nA	2
	treA	2
	tslA	2
	ACC deaminase (VDAG 10392)	1
	Acp1 (PA1869)	1
	AI-2	1
	ampG	1
	ANP1	1
	BbbqrA	1

Table S9 Pathogen-host interaction (PHI) analysis of strain CQ10 (continued)

PHI categories	Genes	Numbers of matched genes in strain CQ10
increased virulence (hypervirulence)	Bsi	1
	cmaA2	1
	croI	1
	Dmt (PD_1542)	1
	EAMY_3003	1
	EAMY_3006	1
	EAMY_3007	1
	EAMY_3008	1
	EAMY_3009	1
	EAMY_3019	1
	EAMY_3021	1
	EAMY_3022	1
	EAMY_3027	1
	EAMY_3201	1
	EAMY_3213	1
	EAMY_3214	1
	EAMY_3216	1
	EAMY_3226	1
	EAMY_3228	1
	essC (lmo0061)	1
	fur	1
	gigX3	1
	gluP (PD_0681)	1
	GroEL	1

Table S9 Pathogen-host interaction (PHI) analysis of strain CQ10 (continued)

PHI categories	Genes	Numbers of matched genes in strain CQ10
increased virulence (hypervirulence)	HmbR	1
	hrcC	1
	HSP90	1
	katG	1
	L7052	1
	mce1R	1
	Mgt2 (CNAG_03502)	1
	modA10	1
	Ndk	1
	Oma1	1
	PA0005	1
	PA0336 (RppH)	1
	PepN	1
	PKR1	1
	pvdA	1
	PXO_00049	1
	raxA	1
	raxP (cysD)	1
	rcsA	1
	rsmS (ECA1172)	1
	rssC	1
	Rv0652 (rplL)	1
	Rv1093 (glyA1)	1
	Rv1837c (glcB)	1

Table S9 Pathogen-host interaction (PHI) analysis of strain CQ10 (continued)

PHI categories	Genes	Numbers of matched genes in strain CQ10
increased virulence (hypervirulence)	secA2	1
	secY	1
	Uge3	1
	UmaA1	1
	vfr	1
	vraS	1
effector (plant avirulence determinant)	BP1026B_II1587	16
	VasH	9
	ACE1	7
	EAMY_3223	6
	LpdA	6
	TssL	5
	EAMY_3024	4
	Hcp	4
	Stk (ORF: z1444)	3
	ClpV	3
	EAMY_3026	2
	EAMY_3220	2
	TssA	2
	HopI1	2
	ipx10	2
	Me23	2
	pagC	2
	pip (proline iminopeptidase)	2

Table S9 Pathogen-host interaction (PHI) analysis of strain CQ10 (continued)

PHI categories	Genes	Numbers of matched genes in strain CQ10
effector (plant avirulence determinant)	sssB	2
	TssC	2
	TssB	1
	TssD	1
	TssE	1
	TssF	1
	TssG	1
	TssJ	1
	TssK	1
	TssM	1
	VasK	1
	avrAC (XopAC)	1
	bsaO	1
	bsaZ	1
	BtpB	1
	EAMY_3211	1
	esx-3	1
	katN	1
	Mg1LysM	1
	Mg3LysM	1
	spaP	1
	sseB	1
	T3SS	1
	VgrG	1

Table S9 Pathogen-host interaction (PHI) analysis of strain CQ10 (continued)

PHI categories	Genes	Numbers of matched genes in strain CQ10
effector (plant avirulence determinant)	XC_3703	1
	xrp8	1
	ysaC	1

Table S10 Carbohydrate active enzymes (CAZymes) analysis of strain CQ10

HMMER	dbCAN_sub	DIAMOND	Categories
GT9(87-308)	GT9_e292	GT9	GTs
GT9(85-322)	GT9_e18	GT9	GTs
GT9(77-300)	GT9_e71	GT9	GTs
GT9(76-316)	GT9_e14	GT9	GTs
GT9(69-305)	GT9_e84	GT9	GTs
GT9(103-331)	GT9_e213	GT9	GTs
GT83(5-520)	GT83_e0	GT83	GTs
GT81(117-322)	GT81_e1	GT81	GTs
GT8(4-251)	GT8_e175	GT8	GTs
GT8(23-268)	GT8_e20	GT8	GTs
GT56(2-348)	GT56_e0	GT56	GTs
GT51(63-226)	GT51_e71	GT51	GTs
GT51(61-225)	GT51_e0	GT51	GTs
GT51(54-229)	GT51_e130	GT51	GTs
GT51(208-378)	GT51_e28	GT51	GTs
GT5(2-474)	GT5_e31	GT5	GTs
GT4(226-371)	GT4_e731	GT4	GTs
GT4(223-374)	GT4_e91	GT4	GTs
GT4(203-336)	GT4_e3707	GT4	GTs
GT4(200-341)	GT4_e1381	GT4	GTs
GT4(170-320)	GT4_e2087	GT4	GTs
GT35(91-798)	GT35_e0	GT35	GTs
GT35(91-798)	GT35_e0	GT35	GTs
GT35(101-810)	GT35_e0	GT35	GTs

Table S10 Carbohydrate active enzymes (CAZymes) analysis of strain CQ10 (continued)

HMMER	dbCAN_sub	DIAMOND	Categories
GT30(32-210)	GT30_e0	GT30	GTs
GT28(183-339)	GT28_e46	GT28	GTs
GT26(62-231)	GT26_e141	GT26	GTs
GT25(2-100)	GT25_e93	-	GTs
GT20(2-457)	GT20_e1	GT20	GTs
GT20(13-449)	GT20_e18	GT20	GTs
GT2(9-133)	GT2	GT2	GTs
GT2(9-118)	GT2	GT2	GTs
GT2(8-128)	GT2	GT2	GTs
GT2(7-127)	GT2	GT2	GTs
GT2(7-119)	GT2	GT2	GTs
GT2(4-166)	GT2	GT2	GTs
GT2(4-166)	GT2	GT2	GTs
GT2(3-99)	GT2	GT2	GTs
GT2(276-447)	GT2+GT2	GT2	GTs
GT2(253-435)	GT2	GT2	GTs
GT2(130-301)	GT2+GT2	GT2	GTs
GT2(11-172)	GT2	GT2	GTs
GT2(10-132)	GT2	GT2	GTs
GT19(8-365)	GT19_e17	GT19	GTs
GT101(55-280)	GT101_e11	GT101	GTs
GT1(8-414)	GT1_e11	GT1	GTs
-	GT4_e3814	GT4	GTs
-	GT4_e3218	GT4	GTs

Table S10 Carbohydrate active enzymes (CAZymes) analysis of strain CQ10 (continued)

HMMER	dbCAN_sub	DIAMOND	Categories
-	GT2	GT2	GTs
-	GT2	GT2	GTs
GH8(27-328)	GH8_e29	GH8	GHs
GH77(146-684)	GH77_e27	GH77	GHs
GH77(141-680)	GH77_e27	GH77	GHs
GH73(762-888)	GH73_e162	GH73	GHs
GH73(165-301)	GH73_e114	GH73	GHs
GH53(37-396)	GH53_e15	GH53	GHs
GH43_26(7-306)	GH43_e105	GH43_26	GHs
GH42(19-391)	GH42_e20	GH42	GHs
GH37(71-546)	GH37_e6	GH37	GHs
GH37(64-540)	GH37_e6	GH37	GHs
GH33(39-376)	GH33_e0	GH33	GHs
GH32(31-336)	GH32_e57	GH32	GHs
GH31(236-682)	GH31_e60	GH31	GHs
GH3(98-319)	GH3_e1	GH3	GHs
GH3(59-283)	GH3_e19	GH3	GHs
GH28(30-357)	GH28_e5	GH28	GHs
GH28(29-400)	GH28_e13	GH28	GHs
GH24(30-161)	GH24_e283	-	GHs
GH24(29-160)	GH24_e190	-	GHs
GH23(61-207)	GH23_e734	GH23	GHs
GH23(485-616)	GH23_e96	GH23	GHs
GH23(298-447)	GH23_e225	GH23	GHs

Table S10 Carbohydrate active enzymes (CAZymes) analysis of strain CQ10 (continued)

HMMER	dbCAN_sub	DIAMOND	Categories
GH23(207-352)	GH23_e150	GH23	GHs
GH23(108-252)	GH23_e819	GH23	GHs
GH20(328-763)	GH20_e0	GH20	GHs
GH20(152-582)	GH20_e52	GH20	GHs
GH2(41-919)	GH2_e92	GH2	GHs
GH15(238-597)	GH15_e9	GH15	GHs
GH15(222-588)	GH15_e13	GH15	GHs
GH13_9(274-574)	GH13_e200	GH13_9	GHs
GH13_5(31-373)	GH13_e245	GH13_5	GHs
GH13_26(25-309)	GH13_e105	GH13_26	GHs
GH13_18(82-422)	GH13_e137	GH13_18	GHs
GH13_11(188-536)	GH13_e48	GH13_11	GHs
GH13_11(172-522)	GH13_e48	GH13_11	GHs
GH13_10(123-436)	GH13_e85	GH13_10	GHs
GH109(1-347)	GH109_e5	-	GHs
GH105(60-376)	GH105_e57	GH105	GHs
GH103(62-351)	GH103_e0	GH103	GHs
GH102(126-259)	GH102_e2	GH102	GHs
GH1(7-465)	GH1_e42	GH1	GHs
GH1(6-476)	GH1_e0	GH1	GHs
GH1(51-345)	GH1_e19	GH1	GHs
GH1(4-472)	GH1_e85	GH1	GHs
GH1(31-357)	GH1_e73	GH1	GHs
GH1(2-459)	GH1_e85	GH1	GHs

Table S10 Carbohydrate active enzymes (CAZymes) analysis of strain CQ10 (continued)

HMMER	dbCAN_sub	DIAMOND	Categories
CBM48(17-107)	CBM48_e3	CBM48	CBMs
CBM48(125-211)	CBM48_e2	CBM48	CBMs
CBM48(10-98)	CBM48_e3	CBM48	CBMs
-	CBM50_e657	CBM50	CBMs
-	CBM50_e567	CBM50	CBMs
-	CBM50_e486	CBM50	CBMs
-	CBM50_e126	CBM50	CBMs
-	CBM48_e0	CBM48	CBMs
AA6(3-195)	AA6_e2	-	AAs
AA3_2(2-532)	AA3_e57	AA3_2	AAs
AA1(66-524)	AA1_e20	-	AAs
-	AA2_e1	AA0	AAs
CE9(5-376)	CE9_e0	CE9	CEs
CE11(4-276)	CE11_e22	CE11	CEs
-	CE4_e177	CE4	CEs

HMMER: the results of the HMMER run versus the dbCAN database. dbCAN_sub: the results of the HMMER run versus the dbCAN-sub database. DIAMOND: the results of the DIAMOND blast versus the CAZy database. GTs: GlycosylTransferases. GHs: Glycoside Hydrolases. CBMs: Carbohydrate-Binding Modules. AAs: Auxiliary Activities. CEs: Carbohydrate Esterases.

Table S11 Biosynthetic gene clusters (BGCs) predicted by antiSMASH 6.1 softwares in the genome of strain CQ10

Region	Location	Type	Comounds	MIBiG reference	Organism	Similarity score
Region 1.1	Chromosome	saccharide	jinggangmycin	BGC0000701.1	*Streptomyces hygroscopicus* subsp. *yingchengensis*	0.61
Region 1.2	Chromosome	RiPP	WGK	BGC0001929.1	*Streptococcus ferus*	0.55
Region 1.3	Chromosome	other	cycloserine	BGC0000896.1	*Streptomyces lavendulae* subsp. *lavendulae*	0.49
Region 1.4	Chromosome	saccharide	O-antigen	BGC0000784.1	*Pseudomonas aeruginosa*	0.58
Region 1.5	Chromosome	NRP	bacillibactin	BGC0001185.1	*Bacillus velezensis* FZB42	0.67
Region 2.1	Plasmid	other	bacilysin	BGC0001184.1	*Bacillus velezensis* FZB42	0.62
Region 4.1	Plasmid	other	desferrioxamine E	BGC0001572.1	*Pantoea agglomerans*	0.90
Region 5.1	Plasmid	terpene	carotenoid	BGC0000638.1	*Pantoea ananatis*	0.85
